# Mitigating the risk of flow deterioration by deferring stent optimization in STEMI patients with large thrombus burden: Insights from a prospective cohort study

**DOI:** 10.1186/s12872-023-03540-0

**Published:** 2023-10-12

**Authors:** Giacomo Maria Cioffi, Yuan Zhi, Mehdi Madanchi, Thomas Seiler, Leah Stutz, Varis Gjergjizi, Jean-Paul Romero, Adrian Attinger-Toller, Matthias Bossard, Florim Cuculi

**Affiliations:** 1 Cardiology Division, Heart Center, Luzerner Kantonsspital, Lucerne, Switzerland; 2 Division of Cardiology, Hamilton General Hospital, Hamilton Health Sciences, Hamilton, McMaster University, Ontario, Hamilton, Canada; 3 Faculty of Health Sciences and Medicine, University of Lucerne, Luzerner Kantonsspital, 6000 Luzern 16, Switzerland

**Keywords:** STEMI, Myocardial infarction, Optical coherence tomography, Stent optimization, No-reflow

## Abstract

**Objectives:**

It is uncertain, if omitting post-dilatation and stent oversizing (stent optimization) is safe and may decrease the risk for distal thrombus embolization (DTE) in STEMI patients with large thrombus burden (LTB).

**Background:**

In patients with ST-segment elevation myocardial infarction (STEMI) undergoing primary percutaneous coronary intervention (pPCI) with stenting, (DTE) and flow deterioration are common and increase infarct size leading to worse outcomes.

**Methods:**

From a prospective registry, 74 consecutive STEMI patients with LTB undergoing pPCI with stenting and intentionally deferred stent optimization were analyzed. Imaging data and outcomes up to 2 years follow-up were analyzed.

**Results:**

Overall, 74 patients (18% females) underwent deferred stent optimization. Direct stenting was performed in 13 (18%) patients. No major complications occurred during pPCI. Staged stent optimization was performed after a median of 4 (interquartile range (IQR) 3; 7) days. On optical coherence tomography, under-expansion and residual thrombus were present in 59 (80%) and 27 (36%) cases, respectively. During deferred stent optimization, we encountered no case of flow deterioration (slow or no-reflow) or side branch occlusion. Minimal lumen area (mm^2^) and stent expansion (%) were corrected from 4.87±1.86mm to 6.82±2.36mm (*p*<0.05) and from 69±18% to 91±12% (*p*<0.001), respectively. During follow-up, 1 patient (1.4%) required target lesion revascularization and 1 (1.4%) patient succumbed from cardiovascular death.

**Conclusions:**

Among STEMI patients with LTB, deferring stent optimization in the setting of pPCI appears safe and potentially mitigates the risk of DTE. The impact of this approach on infarct size and clinical outcomes warrants further investigation in a dedicated trial.

## Introduction

Primary percutaneous coronary intervention (pPCI) with stent implantation has transformed the treatment of acute ST-elevation myocardial infarction (STEMI) and now represents the standard of care for reperfusion in this setting [1, 2].

Although stent implantation has been shown to be superior to plain old balloon angioplasty (POBA) ensuring vessel patency in STEMI setting, it may lead to distal embolization of thrombus (DTE) and subsequent flow deterioration (slow- or no-reflow phenomenon) with microvascular obstruction, which is an established predictor for larger infarction size and worse clinical outcomes [3–5]. Moreover, the incidence of target lesion failure (TLF) due to in-stent restenosis and stent thrombosis (ST) following stent implantation in the STEMI setting remains relatively high (up to 6% after 1 year follow-up), despite the advent of modern drug eluting stent (DES) platforms [6]. Suboptimal stent implantation (e.g. due to stent under-expansion, edge dissection or geographical miss) has been identified as a key factor for TLF among STEMI patients [6, 7]. It is also well established that post-dilatation (stent optimization) and oversizing stents during pPCI bares an increased risk for thrombus embolization and flow deterioration [8, 9]. Despite extensive research, multiple devices and strategies have failed to adequately address the clinical conundrum of DTE in STEMI patients, especially among those with large thrombus burden (LTB).

We have recently published a case series of patients presenting with ST, where we intentionally omitted aggressive stent sizing and post-dilatation in the pPCI setting in order to mitigate the risk of DTE and flow deterioration [10]. In those cases, we encountered no periprocedural flow deterioration and all patients underwent uncomplicated stent optimization using optical-coherence tomography (OCT), as part of a staged procedure, a few days later [10]. Thus, a strategy of pPCI with stent implantation followed by intravascular imaging-guided PCI with post-dilatation (stent optimization) several days later may have the potential to overcome some of the afore discussed challenges.

This study summarizes our early experience, including the procedure-related findings and outcomes, of our STEMI cohort with LTB undergoing pPCI and intentional deferred stent optimization.

## Methods

### Study design

This analysis stems from the ongoing prospective OPTIMISER registry – *A Prospective Cohort Study to Describe the OPTIMal Management and Outcomes of PatIents PreSEnting With Acute MyocaRdial Infarction* (ClinicalTrials.gov Identifier: NCT04988672), which aims to assess procedural characteristics and outcomes of patients undergoing PCI for revascularization in myocardial infarction (MI) with contemporary practice. This prospective cohort study had been established at the Heart Center of the Luzerner Kantonsspital (Lucerne, Switzerland), which is a tertiary cardiology facility of the central Switzerland (annual PCI volume >1700 procedures). This study complies with the Declaration of Helsinki and was approved by the local ethics committee (Ethikkommission Nordwest- und Zentralschweiz (EKNZ) with project ID (2020-02559)). Informed consent was obtained from all patients.

### Study population

We analyzed consecutive patients presenting with STEMI and referred for reperfusion with pPCI, who showed LTB on angiography (Thrombolysis in Myocardial Infarction (TIMI) Thrombus Grade 4 or 5) and among whom stent optimization (e.g. post-dilatation with non-compliant balloons at high-pressure) was omitted to reduce risks of DTE. As part of a staged non-culprit lesion PCI or planned control angiography, all analyzed patients underwent OCT-guided PCI with assessment and optimization (e.g. post-dilatation and/or additional stenting) of their index lesion and other vessels, if necessary.

### Data collection and outcome definitions

Information about patients´ baseline characteristics, including medical history and medication, vital parameters, laboratory values, procedural characteristics, and complications, as well as clinical outcomes were collected using dedicated questionnaires (REDCap, Version 10.6.28, established by the Vanderbilt University, Tennessee, U.S.A.). We collected prospective follow-up information. Clinical follow-up information was obtained from the studied subjects by pre-defined clinic visits or telephone interviews at 30 days, 6 months, 1 year and 2 years after the index procedure. All outcomes were independently reviewed. Our clinical endpoints of interest included among others major adverse cardiac and cerebrovascular event (MACCE) defined as composite of cardiovascular death, clinically driven target lesion revascularization (TLR), target vessel myocardial infarction (TV-MI) and stroke [11]. Cardiac death, clinically driven TLR and ST were defined as suggested by the *Academic Research Consortium (ARC)* criteria [12, 13]. For MI, we applied the universal definition [11]. ST was classified as definite, probable, and possible [12, 13].

### Management and procedural characteristics

Management of STEMI patients complied with the latest European society of cardiology (ESC) guidelines [14, 15]. All patients were pre-treated with aspirin (250-500mg intravenously) if tolerated. Additionally, the patients received a bolus of 5000 IU of unfractionated heparin. A loading dose of ticagrelor (180 mg) was given as part of pre-treatment prior to the urgent angiography and pPCI in STEMI patients. Otherwise, the second antiplatelet agent, namely ticagrelor (180 mg), prasugrel (60 mg) or clopidogrel (600 mg), was administered following angiography and prior to PCI. In selected high-risk cases or patients unable to ingest an oral ADP receptor antagonist, cangrelor was administered intravenously during the procedure. Glycoprotein receptor (GP) IIb/IIIa antagonists were only administered in selected cases (e.g. patients lacking pretreatment with antiplatelets). For pPCI procedure, we usually administered unfractionated heparin (80-100 IU/kg body weight). As per our institutional recommendations, we generally prescribed therapeutic dosages of unfractionated heparin for at least 24 to 48 hours following pPCI in MI cases with LTB. After pPCI, guideline-based dual antiplatelet therapy (DAPT) was prescribed for at least 12 months. In patients requiring anticoagulation, initial triple therapy for 7-28 days was followed by clopidogrel and a direct oral anticoagulant for 12 months [16].

At our institution, pPCI in the STEMI setting generally involves pre-dilatation using a small-sized (1.5-2.0 mm diameter) semi- or non-compliant balloon followed by implantation of a latest generation drug eluting stent (DES). Direct stenting is only performed in selected cases and thrombectomy is usually performed as a bailout strategy or in cases with very LTB.

**Fig. 1:**
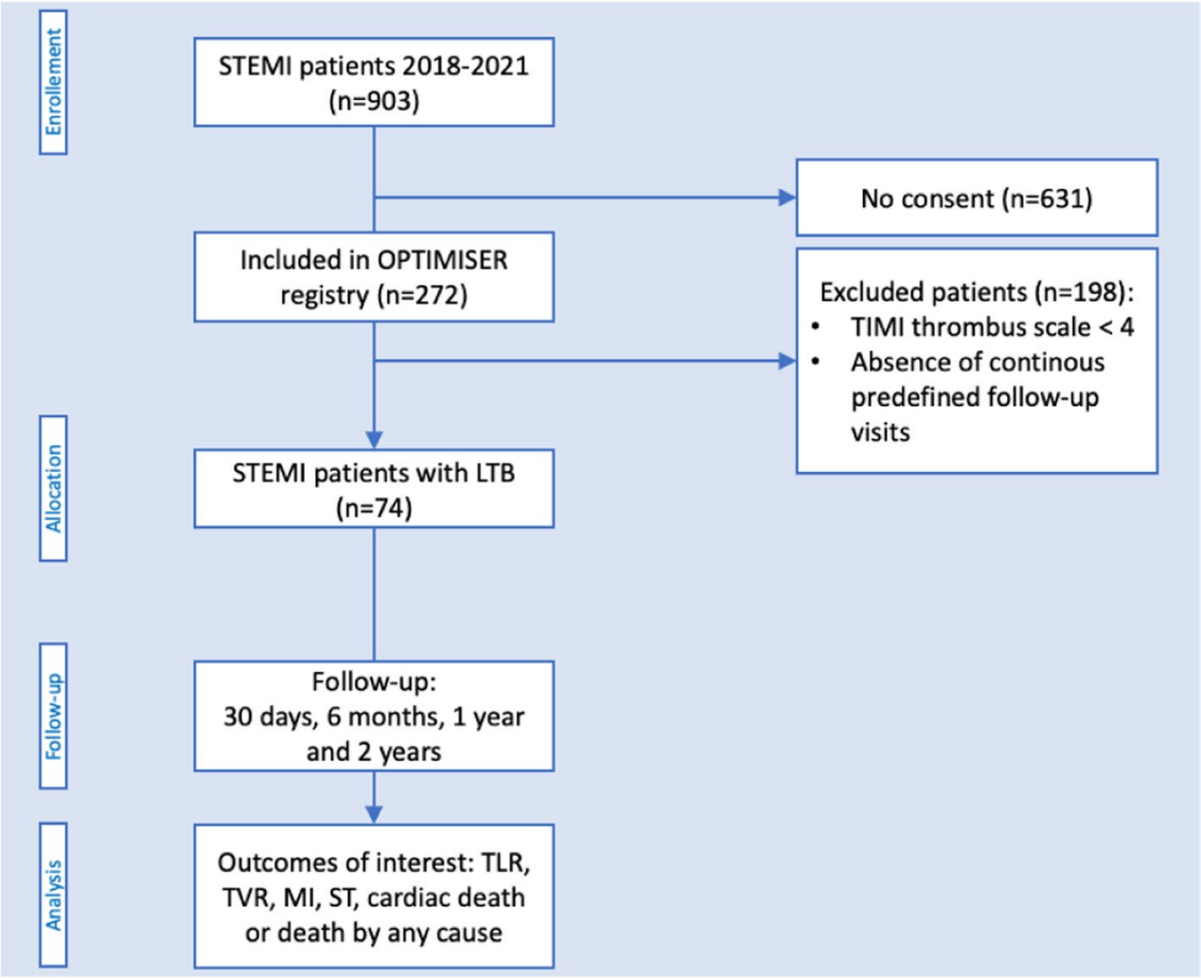
Study flow chart. LTB = large thrombus burden; MI = myocardial infarction; TLR = target lesion revascularization; TVR = target vessel revascularization; TLF = target lesion failure; STEMI = ST-segment myocardial infarction; ST = Stent thrombosis

In STEMI patients with angiographic evidence of LTB (TIMI Thrombus Scale 4 or 5), we generally avoid oversizing of stents, we preferably implant stent devices at low pressure (e.g. nominal pressure) and omit or limit post-dilatation involving large-sized balloons (“stent optimization”), expecting a lower risk of DTE during pPCI, as illustrated in Fig. 1. It is important to mention that some post-dilatation during pPCI might become necessary in the following scenarios: (I) cases involving bifurcations, very large or aneurysmatic vessels, where operator encounter massive undersizing at the proximal end of the stented segment, which could potentially impair rewiring and crossing with PCI devices. In those cases post-dilatation actually reflects a kind of proximal optimization technique (POT) facilitating rewiring and/ or recrossing; (II) cases with very restrictive lesions (e.g. heavily fibrocalcific lesions), among which the stent remains severely underexpanded and leaving the stent like that behind, could potentially put the patient at risk for early stent thrombosis; (III) and cases where it is necessary to rewire and fenestrate important side branches. In cases undergoing staged stent-optimization, we reassessed the culprit vessel as part of the staged PCI procedure of a non-culprit lesion or planned control (follow-up) angiography after the index PCI. As part of our clinical routine, we commonly use OCT for staged PCI procedures.

### Angiographic analysis

All coronary angiograms (CA) were analyzed with a dedicated software package (Intellispace cardiovascular, Phillips, Koninklijeke, Netherlands) by two independent physicians, who were not involved in the PCI procedures (GMC and MM). The lesions were classified according to the ACC/AHA lesion classification [17]. Bifurcation lesions were categorized according Medina classification [18]. Thrombus burden was analyzed and defined based on the TIMI thrombus grading [19]. Dissections were classified according to the National Heart, Lung and Blood Institute (NHLBI) classification system for intimal tears, consisting of Type A through Type F [20].

### OCT acquisition and analysis

OCT investigations were acquired, when feasible before and after PCI. For OCT, we used the Optis Ilumien^™^ system and the Dragon Fly Duo OCT Imaging Catheter (Abbott Vascular, Santa Clara, CA) with motorized pullback (25 mm/s) using a non-occlusive flushing technique according to manufacturer’s recommendations. Images of the culprit segment and of the reference segments 5 mm proximal and distal of the previously implanted stent devices were acquired. OCT pullbacks were registered and assessed offline using dedicated software stations (OPTIS™ Imaging Software, Abbott Vascular, Santa Clara, CA). We applied the same methodology and definitions, as described elsewhere earlier [13].

### Statistical analysis

Statistical analyses were primarily descriptive. Categorical variables are displayed as numbers and percentages, and continuous variables are presented as means (±standard deviations, SD) or medians (interquartile ranges, IQR), as appropriate. Time-to-event analyses after the index procedure were estimated with the Kaplan-Meier method. A *p*-value <0.05 was considered statistically significant. Statistical analyses were performed with STATA 17 (StataCorp LCC, Lakeway Drive, Texas, USA).

## Results

### Study population

**Fig. 2:**
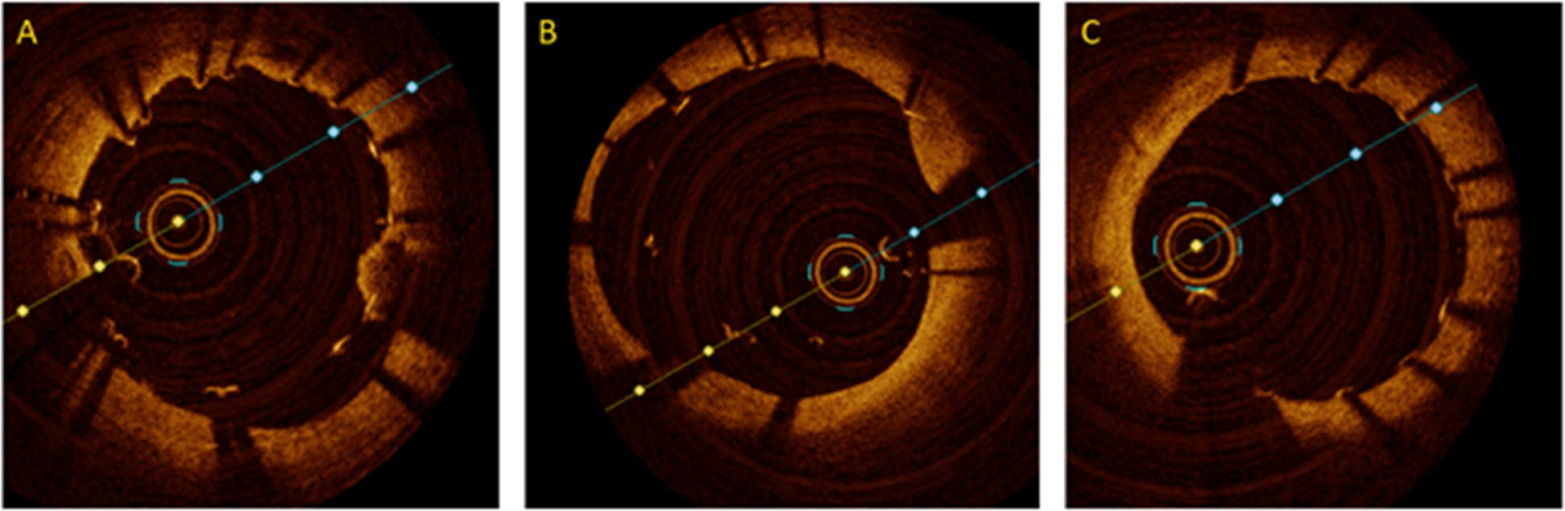
OCT findings of a STEMI patient undergoing treatment of proximal RCA. Optical coherence images (OCT) images of a STEMI patient who had suffered from a thrombotic occlusion of the proximal to mid RCA: (**A**) OCT of the culprit segment at the end of the index procedure, showing underexpansion and residual thrombus between vessel and stent and intrastent. **B** OCT at staged PCI (after 4 days) before stent-optimization, showing no residual thrombus, but significant underexpansion and malapposition. **C** Final OCT run after stent optimization with non-compliant balloons (post-dilatation up to 25 atm)

The study flow-chart is presented in Fig. 2. Overall, we analyzed 74 STEMI patients with LTB who underwent pPCI with an intention for deferred stent optimization approach, who have been treated at our institution between 2018 and 2021. The mean age was 62±9 years and the majority (82%) were males. The initial mean left ventricular ejection fraction (LVEF) at baseline was 39±11%, 15 (20%) patients were resuscitated prior to hospital admission, and 16 (21%) patients were in cardiogenic shock requiring mechanical hemodynamic support. Further details on baseline characteristics are displayed in Table 1.

**Table 1:** Baseline characteristics

	***N° of patients*** *(n* = *74)*
Age, years ± SD	62 ± 9
Males, n (%)	61 (82)
BMI, Kg/m^2^ (IQR)	26 (24; 28)
Killip Class, n (%)	
I	53 (72)
II	4 (5)
III	1 (1)
IV	16 (21)
Resuscitation prior to hospital admission, n (%)	15 (20)
Troponin T (Peak), ng/L (IQR)	4528 (1939; 10,438)
Peak CK-MB, U/L (IQR)	210 (79; 310)
Initial LV-EF (%)	39 ± 11
Duration of ICU stay, days (IQR)	1 (1; 3)
Duration of hospitalization, days (IQR)	6 (4; 10)
Mechanical support, n (%)	
Impella CP	15 (20)
IABP	3 (4)
Arterial hypertension, n (%)	37 (50)
Diabetes mellitus, n (%)	16 (22)
Dyslipidemia, n (%)	46 (62)
Current smoking, n (%)	33 (45)
Family history of premature CAD, n (%)	16 (22)
Previous MI, n (%)	7 (10)
Previous CABG, n (%)	1 (1)
History Of HFrEF, n (%)	1 (1)
Antithrombotics (post-PCI), n (%)	
Aspirin	72 (97)
Clopidogrel	4 (5)
Ticagrelor	55 (74)
Prasugrel	15 (20)
Direct oral anticoagulant	4 (4)
Gp IIb/IIIa antagonists	23 (31)

Data are mean (*SD* Standard deviation), median (*IQR* Interquartile range) or number (percentage), as appropriate. *BMI* Body mass index, *STEMI* ST-segment elevation myocardial infarction, *CK-MB* Creatine kinase myocardial band, *LV-EF* Left ventricular jection fraction, *ICU* Intensive care unit, *IABP* Intra-aortic balloon pump, *CAD* Coronary artery disease, *MI* Myocardial infarction, CABG Coronary artery bypass grafting, *HFrEF* Heart failure with reduced ejection fraction, *PCI* Percutaneous coronary intervention, *GP IIb/IIIa* Glycoprotein IIb/IIIa

### Lesion and procedural characteristics

The most frequent culprit vessel was the left anterior descending (LAD) artery (47%), followed by the right coronary artery (RCA) (38%). In 9 (12%) patients, the STEMI was caused by ST. Of note, 54 (73%) patients presented with TIMI thrombus grade 5. Direct stenting was performed in 13 (18%) patients. We did not observe any no-reflow phenomenon or acute vessel closure during pPCI.

Staged PCI with stent optimization occurred on median 4 (IQR 3; 7) days after the index procedure. For stent optimization, non-compliant balloons were mostly used (88%) with a mean post-dilatation pressure of 20 (IQR 16; 25) atm. Totally, 3 non-flow limiting dissections (Type B and C) were found, none of them requiring additional stent placement. Further details about lesion and procedural characteristics are reported in Tables 2 and 3.

**Table 2:** Angiographic and procedural characteristics: (A) at index procedure; and (B) at staged procedure

***Primary PCI procedure***	***N° of patients*** *(n* = *74)*
(**A**)	
Access (%)	
Radial	61 (82)
Femoral	13 (18)
Duration of procedure (min)	31 (24; 47)
Contrast media (ml)	149 (109; 189)
Culprit vessel (%)	
Left main	2 (3)
Left anterior descending	35 (47)
Left circumflex	9 (12)
Right coronary artery	28 (38)
Complete ST-segment resolution (%)	39 (53)
Initial TIMI flow (%)	
0	62 (84)
1	11 (15)
2	1 (1)
Stent thrombosis (%)	9 (12)
Bifurcation lesions (%)	31 (40)
Aorto-ostial lesions (%)	8 (12)
Degree of calcification (%)	
None/mild	51 (69)
Moderate	20 (27)
Severe	5 (7)
Length more than 20 mm (%)	60 (86)
Thrombectomy (%)	
Primary	18 (25)
Bailout	6 (8)
Pre-dilatation (%)	61 (82)
Direct stenting (%)	13 (18)
Pre-dilatation device (%)	
SC balloon	18 (24)
NC balloon	48 (65)
Stent type used (%)	
Permanent polymer-based DES	68 (92)
Bioregredable polymer-based DES	6 (8)
No. of stent used (%)	
1	54 (73)
2	17 (24)
≥ 3	3 (3)
Mean device diameter (mm)	3.4 ± 0.35
Total device length (mm)	36 ± 17
Deployment pressure (atm)	12 ± 4
Post-dilatation (%)	22 (29)
Post-dilatation device (%)	
vSC balloon	6 (8)
NC balloon	32 (43)
Maximal post-dilatation pressure (atm)	18 ± 7
Final TIMI flow (%)	
0	-
1	-
2	5 (6)
3	69 (94)
Periprocedural complications (%)	
VF/VT during pPCI	7 (9)
(**B**)	
***Staged PCI procedure***	***N° of patients*** *(n* = *74)*
Duration of procedure (min)	42 (32; 64)
Contrast media, ml (IQR)	174 (119; 236)
Optimized vessels (%)	
Left main	3 (4)
Left anterior descending	34 (46)
Left circumflex	9 (12)
Right coronary artery	29 (39)
Angiographic evidence of thrombus (%)	2 (3)
Post-dilatation device (%)	
SC balloon	8 (11)
NC balloon	65 (88)
OPN balloon	12 (16)
Number of post-dilatation balloons (%)	
1	39 (54)
2	26 (36)
≥ 3	9 (12)
Mean size (mm)	3.5 (3.5; 4)
Post-dilatation pressure (atm)	20 (16; 25)
Secondary PCI related complications (n)	
Edge Dissection	3 (4)

Data are mean (*SD* Standard deviation), median (*IQR* Interquartile range) or number (percentage), as appropriate. *PCI* Percutaneous coronary intervention, *TIMI* Thrombolysis in Myocardial Infarction, *DES* Drug eluting stent, *SC* Semi compliant, *NC* Non-compliant, *pPCI* Primary *PCI* OCT Optical coherence tomography, *VF* Ventricular fibrillation, *VT* Ventricular tachycardia

**Table 3:** Findings on optical coherence tomography at the deferred stent optimization procedure of the index lesion

*(n* = *74)*	*Before* *optimization*	*After* *optimization*	*P-value*^*†*^
OCT findings, n (%)			
Thrombus in native vessel	23 (31)	16 (22)	0.08
Thrombus in stent	27 (36)	19 (26)	< 0.05
Underexpansion	59 (80)	5 (7)	< 0.05
Edge dissection	1 (1)	3 (4)	0.84
Mean reference vessel diameter (mm)	3.10 ± 0.52	3.34 ± 0.53	< 0.05
Residual DS (%)	23 ± 12	16 ± 14	< 0.05
MSA (mm^2^)	5.54 ± 1.96	8.07 ± 2.28	< 0.05
Mean SE (%)	69 ± 18	91 ± 12	< 0.05
Geographical miss, n (%)	6 (8)	-	-
Thrombus burden, n (%)			
None	24 (32)	39 (53)	
Small (< 90° / 1 quadrant)	29 (39)	27 (36)	
Medium (< 180° / 2 quadrants)	17 (23)	7 (9)	
Large (> 180° / > 2 quadrants)	4 (5)	1 (1)	
Malapposition, n (%)			
No (0–200 µm)	6 (8)	53 (72)	
Minor (200–300 µm)	7 (9)	10 (14)	
Major (> 300 µm)	61 (82)	1 (1)	
Dissection, n (%)			
No	-	53 (72)	
Minor	-	19 (26)	
Major	-	1 (1)	
Intramural hematoma	-	1 (1)	

Data are mean (*SD* Standard deviation), median (*IQR* Interquartile range) or number (percentage), as appropriate. *DS* Diameter stenosis, *MLA* Minimal lumen area, *MLD* Minimun lumen diameter, *MSA* Mean surface area; Optical coherence tomography; *SE* Stent expansion

^a^A median of 4 (3;7) days has elapsed since the index procedure (pPCI) and 2 (2; 3) OCT runs were obtained per patient and lesion

^†^ P-values were based on student’s t-tests, Fisher’s test, Mann–Whitney U- tests, Chi-square tests or McNenam tests, as appropriate

### OCT findings at follow-up

At staged PCI (deferred stent optimization) procedure, OCT was performed in all 74 (100%) patients. Generally, 2 runs (IQR 2; 3) were obtained per lesion. Before optimization, underexpansion 59 (80%), major malapposition 61 (82%) and thrombus in stent 27 (36%) were the main findings. Of these, thrombus burden was either medium or large in 17 (23%) or 4 (5%) cases, respectively. After stent-optimization it reduced to 7 (9%) and 1 (1%), respectively. Geographical miss was present in 6 cases (8%) and required additional stent implantation. Mean minimal stent area (MSA, mm^2^) significantly increased after stent-optimization from 5.54±1.96mm^2^ to 8.07±2.28mm^2^ (*p* <0.05) as well as stent expansion (SE, %), which increased from 69±18% to 91±12%. Further detailed OCT findings are summarized in Table 3.

### Clinical outcomes

We conducted pre-defined follow-up at 30 days, 6 months, 1 year and 2 years. No patient has been lost during follow-up. We observed a total of 2 (3%) MACCE, 1 (1.4%) patient presented ischemia driven symptoms due to TLR and 1 (1.4%) patient died from a cardiovascular death by a new MI of a non-target vessel. Further details about clinical outcomes are summarized in Table 4.

**Table 4:** Clinical outcomes up to 2 years follow-up

*(n* = *74)*	*30 days*	*6 months*	*1 year*	*2 years*
MACCE^a^, n (%)	-	-	-	2 (2.8)
TLR	-	-	-	1 (1.4)
TV-MI	-	-	-	-
Cardiovascular death	-	-	-	1 (1.4)
Stroke	-	-	-	-
Non-cardiac death, n (%)	-	1 (1.4)	-	1 (1.4)
Minor bleeding, n (%)	2 (3)	-	-	-
Major bleedings (BARC type 3), n (%)^b^	4 (5)	1 (1.4)	-	-
Unplanned hospitalization, n (%)	3 (4)	7 (9.5)	3 (4)	3 (4)
Dyspnea (NYHA Class), n (%)				
II	18 (24)	20 (27)	8 (11)	14 (19)
III	-	-	-	-
Angina (CCS class), n (%)				
No angina	71 (96)	72 (97)	72 (97)	72 (97)
I	2 (3)	1 (1.4)	1 (1.4)	1 (1.4)
II	1 (1.4)	-	-	-
III		1 (1.4)	1 (1.4)	1 (1.4)
LV-EF (%)	51 ± 7	54 ± 9	55 ± 9	53 ± 10

Data are mean (*SD* Standard deviation), median (*IQR* Interquartile range) or number (percentage), as appropriate. *TLR* Target lesion revascularization, *TV-MI* Target vessel myocardial infarction, *BARC* Bleeding Academic Research Consortium, *VARC*  Valve Academic Research Consortium, *LV-EF* Left ventricular ejection fraction, *NYHA* New York Heart Association, *CCS* Canadian cardiovascular society, *MI *Myocardial infarction, *TVR* Target vessel revascularization, *TLF* Target lesion failure

^a^MACCE (Major adverse cardiac and cerebrovascular events) represents a hierarchical composite of cardiovascular death, clinically driven target lesion revascularization (*TLR*), target vessel myocardial infarction (TV-MI) and stroke

^b^Regarding the major bleedings, we observed 1 (1.4%) access (groin) bleeding, 1 (1.4%) skin bleeding and 4 (5.4%) urogenital bleedings during follow-up

## Discussion

Despite optimized medical and procedural management, including use of potent antithrombotics, DTE leading to microvascular obstruction and increased infarction size, still represents a major challenge in pPCI among STEMI patients. Stent oversizing and post-dilatation represent major risk factors for DTE in those patients.

PPCI in STEMI patients with LTB bears the risk of distal embolization, which is associated with larger infarct size and worse clinical prognosis [5, 21, 22]. Over the last decades, multiple drugs (e.g adenosine, GP IIb/IIIa-antagonists) (23, 24) and devices (e.g thrombectomy, sonothrombolysis) [25–30] have shown limited efficacy in mitigating the risk of slow- and no-reflow following pPCI with stent implantation.

**Fig. 3:**
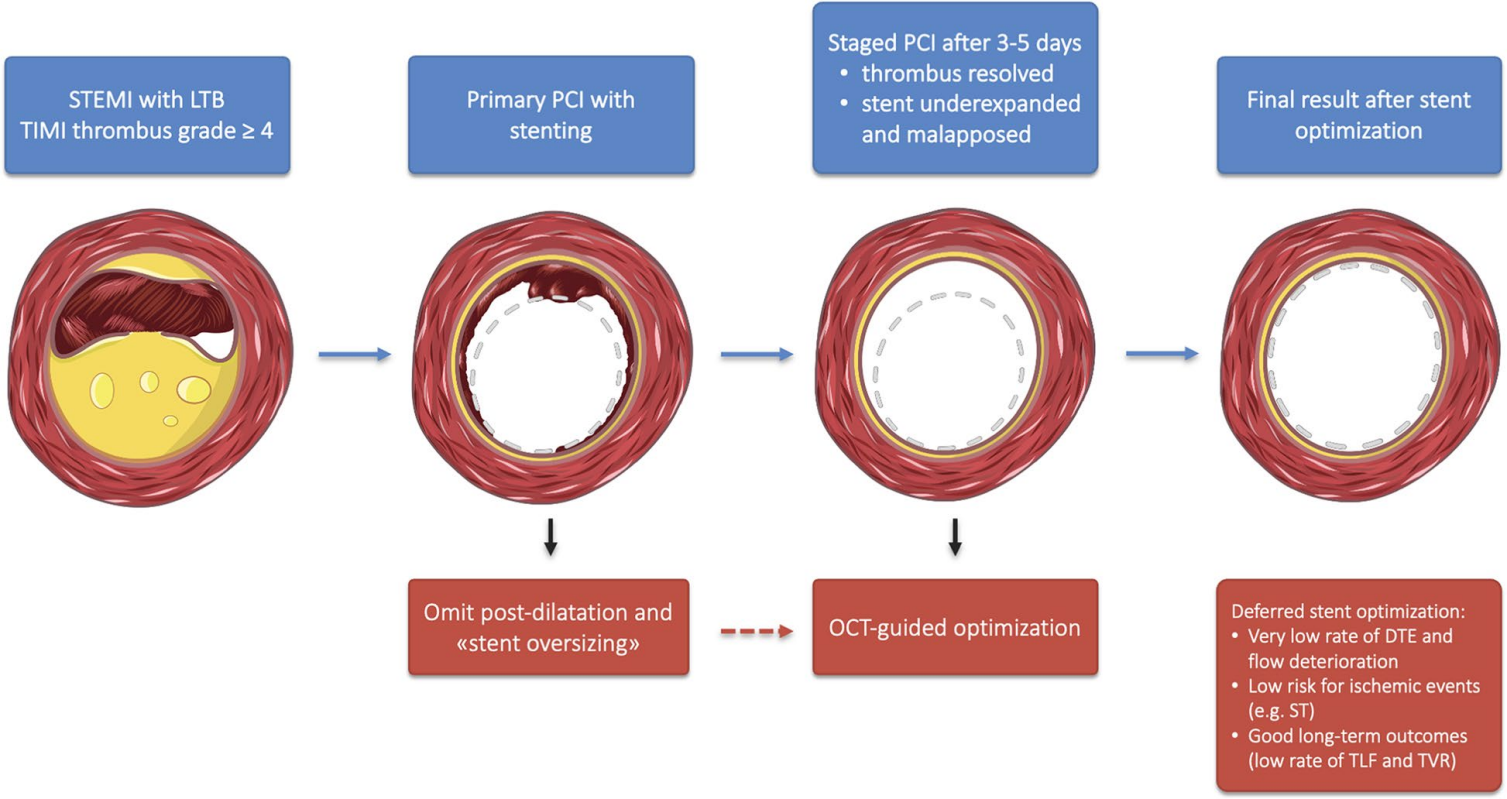
Deferred stent optimization in STEMI patients with large thrombus burden. DTE = distal thrombus embolization; LTB = large thrombus burden; MI = myocardial infarction; OCT = optical coherence tomography; PCI = percutaneous coronary intervention; TIMI = thrombolysis in myocardial infarction; TLR = target lesion revascularization; TVR = target vessel revascularization; TLF = target lesion failure; STEMI = ST-segment myocardial infarction; ST = Stent thrombosis

Here, we are describing a new approach, which postpones stent optimization in STEMI patients with LTB. Two important conclusions can be drawn from this manuscript: Firstly, implanting stents at low (e.g. nominal) pressure in this setting and omitting post-dilatation appears safe, as no thrombotic complications post pPCI were observed. By applying this strategy, the risk for flow deterioration – “slow-flow or no-reflow” – might be significantly reduced. Secondly, our described approach seems furthermore to be associated with favorable long-term clinical outcomes. The main findings are presented in Fig. 3.

The concept of combining pPCI with a second intervention to avoid distal embolization is not new. Knowing that no-reflow is closely related to stent implantation, four randomized trials with a total of 1570 patients have been carried out, namely DEFER-STEMI, MIMI, DANAMI-3 and INNOVATION [31–34]. Of them, only the relatively small DEFER-STEMI trial showed significant reduction of slow reflow and a larger amount of myocardium salvage on cardiac magnetic resonance (CMR), but it did not translate into improved clinical outcomes [31]. Finally, a comprehensive meta-analysis summarizes that a deferred‐stenting strategy, compared with immediate stenting, did not reduce the occurrence of no‐ or slow‐reflow, myocardial infarction, repeat revascularization or death among STEMI patients [35]. But interestingly enough, this analysis also indicated an improved left ventricular function in the long term among STEMI patients managed with the deferred‐stenting strategy. This may again underscore the detrimental impact that aggressive stent implantation in pPCI can have.

So far, one needs to consider that there is a fundamental difference between our approach and the strategy of “delayed or deferred stenting” in the STEMI setting. Stenting has been shown highly effective as a reperfusion strategy in STEMI patients since it compresses the thrombotic material, which in turn sufficiently clears the arterial lumen to restore coronary blood flow. However, by oversizing stents or by post-dilating aggressively in the presence of thrombotic material, thrombotic debris can be sent downstream resulting in microvascular obstruction and no-reflow, which has a devastating impact on infarct size and can hardly be treated.(36) By omitting stent oversizing and postponing stent optimization in STEMI cases with LTB, we did not encountered a single case of no-reflow and only a very small number of our patients had a final TIMI 2 flow. Although at the repeat procedure, the previously implanted stents were substantially underexpanded and malapposed, we did not observe any acute ST, which seems reassuring and may underscores the efficiency of the antiplatelet therapy with potent P2Y12 inhibitors and therapeutic doses of unfractionated heparin.

Long-term results were excellent in this STEMI cohort with a very low rate of TLR (<2%) after two years of follow-up. This might be attributable to the use of intravascular imaging for stent optimization in all patients. We consequently corrected stent underexpansion with aggressive post-dilatation, also including ultra-high-pressure balloons if necessary. Additionally, malapposition was addressed by using semi-compliant and appropriately sized balloons and geographical miss was corrected by implanting additional stents. Globally, intravascular imaging is still underutilized, especially among STEMI patients.(37) But there is mounting evidence, which supports its use in PCI and maybe our study is a call for secondary stent optimization incorporating intravascular imaging in selected STEMI patients.

This study has several important limitations, which need to be considered: (I) The observational character does not permit drawing any firm inferences. (II) Since this analysis derives from a single center registry, generalizability may be limited. (III) Our study lacks a group. Therefore, more prospective, and comparative data is required to assess the utility of the studied approach. (IV) We cannot completely rule out some selection bias due to the lack of a prespecified treatment protocol. (V) Finally, it is important to note that the enrollment rate into the OPTIMISER registry was rather low during the early phase of the study, which could have adversely impacted the diversity of our STEMI cohort and moreover the results.

We acknowledge that any staged angiogram and PCI procedure – as proposed in our approach involving deferred stent optimization in STEMI patients – involves additional costs and some inbound procedure-related risks for a patient. The risks, costs, and potential benefits of additional procedures in MI patients should be carefully weighed against each other. However, one might also need to consider that any (periprocedural) DTE in MI patients causing relevant flow deterioration (slow or no-reflow phenomenon) is inevitably bound to a larger infarct size, which is associated with worse outcomes, including higher risk for heart failure and even death.(38) Furthermore, up to 60% of all STEMI patients suffer from multivessel disease and require additional revascularization procedures. Thus, deferred stent optimization of the previous culprit lesion might be scheduled as part of the staged PCI procedure.

There is a need for novel and enhanced strategies to reduce the risk of flow deterioration following reperfusion among STEMI patients. The strategy of deferred stent optimization represents a radical change from the standard of care and could provide an efficient strategy to minimize DTE in STEMI patients. We are aware that it will require more compelling evidence to introduce a paradigm shift for PCI strategy among STEMI patients. Nonetheless, we are convinced that our study points towards an interesting direction. Since there was no prohibitive safety signal, we firmly believe that our described approach – omitting stent optimization during pPCI – should be further investigated in a dedicated randomized trial.

## Conclusions

Among a selected STEMI patients with LTB, deferring stent optimization in the setting of pPCI appears safe and could moreover help to mitigate the risk of DTE and flow-deterioration associated with stent implantation. The impact of such strategy on infarct size and other clinical outcomes warrants further investigation in a dedicated trial.

## Data Availability

The dataset generated and analyzed during the current study is not publicly available due to legal reasons. Patient informed consent forms do not allow the data to be made publicly but are available from the corresponding author on reasonable request.

## Abbreviations

DAPT: Dual antiplatelet therapy
DES: Drug eluting stents
DTE: Distal thrombus embolization
GP: Glycoprotein receptor
IQR: Interquartile range
LAD: Left anterior descending
LTB: Large thrombus burden
MACCE: Major adverse cardiac and cerebrovascular event
MI: Myocardial infarction
MLA: Minimal lumen area
MSA: Mean surface area
OCT: Optical coherence tomography
PCI: Percutaneous coronary intervention
pPCI: Primary percutaneous coronary intervention
POBA: Plain old balloon angioplasty
RCA: Right coronary artery
SE: Stent expansion
SD: Standard deviation
ST: Stent thrombosis
STEMI: ST-segment elevation myocardial infarction.
TIMI: Thrombolysis in myocardial infarction
TLF: Target lesion failure
TLR: Target lesion revascularization
TV-MI: Target vessel myocardial infarction
TVR: Target lesion revascularization
